# Sotalol does not interfere with the antielectroshock action of selected second-generation antiepileptic drugs in mice

**DOI:** 10.1007/s43440-020-00210-2

**Published:** 2021-01-25

**Authors:** Kinga K. Borowicz-Reutt, Monika Banach, Monika Rudkowska, Anna Stachniuk

**Affiliations:** 1 Independent Unit of Experimental Neuropathophysiology, Medical University of Lublin, Jaczewskiego 8b, PL-20-954, Lublin, Poland; 2 Department of Pathophysiology, Medical University of Lublin, Jaczewskiego 8b, PL-20-954, Lublin, Poland

**Keywords:** Sotalol, Second-generation antiepileptic drugs, Pharmacodynamic interactions, Pharmacokinetic interactions, Electroshock maximal

## Abstract

**Background:**

Due to blocking β-receptors, and potassium KCNH2 channels, sotalol may influence seizure phenomena. In the previous study, we have shown that sotalol potentiated the antielectroshock action of phenytoin and valproate in mice.

**Materials and methods:**

As a continuation of previous experiments, we examined the effect of sotalol on the action of four chosen second-generation antiepileptic drugs (oxcarbazepine, lamotrigine, pregabalin, and topiramate) against the maximal electroshock in mice. Undesired effects were evaluated in the chimney test (motor impairment) and step-through passive-avoidance task (long-term memory deficits). Finally, brain concentrations of antiepileptics were determined by fluorescence polarization immunoassay, while those of sotalol by liquid chromatography–mass spectrometry.

**Results:**

Sotalol at doses of up to 100 mg/kg did not affect the electroconvulsive threshold. Applied at doses of 80–100 mg/kg, sotalol did not affect the antielectroshock action of oxcarbazepine, lamotrigine, pregabalin, or topiramate. Sotalol alone and in combinations with antiepileptics impaired neither motor performance nor long-term memory. Finally, sotalol significantly decreased the brain concentrations of lamotrigine and increased those of oxcarbazepine and topiramate. Pharmacokinetic interactions, however, did not influence the final antielectroshock effects of above-mentioned drug combinations. On the other hand, the brain concentrations of sotalol were not changed by second-generation antiepileptics used in this study.

**Conclusion:**

Sotalol did not reduce the antielectroshock action of four second-generation antiepileptic drugs examined in this study. Therefore, this antidepressant drug should not interfere with antiseizure effects of lamotrigine, oxcarbazepine, pregabalin, and topiramate in patients with epilepsy. To draw final conclusions, our preclinical data should still be confirmed in other experimental models and clinical conditions.

## Introduction

Nowadays, three generations of antiepileptic drugs are already available for the treatment of epilepsy. In contrast to first-generation antiepileptics widely used in monotherapy, newer antiseizure medications are mostly indicated for adjunctive polytherapy. In general, the newer drugs are intended for use in partial onset seizures or primary generalized tonic–clonic seizures and in refractory epilepsy, including Lennox–Gastaut syndrome. Numerous non-epileptic indications further expand the group of patients treated with these medications. In detail, lamotrigine can be used to treat bipolar depression. Its off-label uses also include migraine or panic and binge eating disorders. Pregabalin is indicated for the treatment of neuropathic pain related to diabetes mellitus, spinal cord injury, postherpetic neuralgia, and fibromyalgia. Additional uses include anxiety, bipolar disorder, insomnia, and chronic pain. Finally, topiramate is used in the prophylaxis of migraine while oxcarbazepine (as an off-label drug) in bipolar disorder [1].

Such a wide range of applications increases the likelihood of polypragmasy with second-generation antiseizure drugs. Moreover, the fact that epilepsy is often comorbid with arrhythmias, mostly atrial fibrillation, sudden cardiac arrest, bundle branch block, and ventricular tachycardia is worth emphasizing. In addition, severe cardiac arrhythmias and asphyxia are considered to be the main cause of sudden unexpected death in epilepsy (SUDEP) [2, 3]. Due to the risk of unfavorable interactions, the proper selection of drugs for polytherapy is undoubtedly essential. Cognitive impairment is most observed in combination therapy with antiseizure drugs. Furthermore, some antiepileptics, for instance topiramate, have a greater tendency to cause adverse effects when used in adjunctive therapy than in monotherapy. However, the appropriate choice of drugs seems to be of the highest importance in the combination therapy with antiepileptic and antiarrhythmic drugs. Despite all the differences between electrophysiology of the heart and brain action potentials, the ionic channels expressed in the two organs may be affected by both groups of medications. Therefore, phenytoin is classified as an antiepileptic and antiarrhythmic drug. Furthermore, phenytoin, carbamazepine, and lamotrigine, especially at high doses, can prolong PR and QT intervals leading to arrhythmogenic effects. Antiarrhythmic drugs (e.g., lidocaine and mexiletine) can exhibit antiseizure effects, but may be proconvulsant in overdose [4]. According to some authors, the first-generation antiseizure drugs should be avoided in patients with arrhythmias because of their arrhythmogenic and enzyme-inducing properties. Enhanced metabolism can reduce the effectiveness of antiarrhythmic medications [5]. Sotalol, a class III antiarrhythmic drug, is still used for the treatment of atrial and ventricular tachyarrhythmia in preference to β-blockers, even in patients with ischemic heart disease or pregnant women, if necessary [6–8]. Like other antiarrhythmic agents (amiodarone, propafenone, or flecainide), the therapy with sotalol can be complicated by its possible proarrhythmic action, like torsade de pointes. According to the data available in the literature, women may be particularly sensitive to sotalol-induced delay in cardiac repolarization [9], which should be taken into clinical considerations. However, careful ECG monitoring and QT interval assessment are recommended in each case of antiarrhythmic therapy. Occasionally, cardiac arrhythmia, e.g., above-mentioned torsade de pointes, may manifest as non-epileptic seizures that should be differentiated from epilepsy [10]. On the other hand, in the group of β-blockers, true seizures have been observed only in the case of severe propranolol intoxication [11]. Irrespective of all the undesired effects caused, sotalol is quite frequently used in clinical practice and, for this reason, may interact with simultaneously administered medications. The probability of interactions with antiepileptic drugs is particularly high due to similarity of their mechanisms of action.

The encouraging results of the previous study (sotalol enhanced the antielectroshock action of phenytoin and valproate) and growing interest in neuro-arrhythmology [12] convinced us to continue this research and to evaluate adverse effects in terms of motor and long-term memory impairment. Additionally, brain concentrations of sotalol and antiepileptic drugs were measured to determine the contribution of pharmacokinetic events in the drug interactions revealed.

## Material and methods

### Animals

All study experiments were carried out on 20–25 g female Swiss mice, always at the same time of the day between 9 a.m. and 2 p.m. Experimental groups contained 8–10 animals bred under standard ethical conditions (spacious colony cages with free access to tap water and food, constant temperature and humidity, and natural dark–light cycle). All procedures were approved by the Local Ethical Committee for Animal Experiments (consent No. 66/2016).

### Drugs

Sotalol (SOT), an antiarrhythmic drug, and four second-generation antiepileptic medications, i.e., oxcarbazepine (OXC), lamotrigine (LTG), pregabalin (PGB), and topiramate (TPM), were used in the study. Sotalol was purchased from Sigma, St. Louis, MO, USA, while oxcarbazepine (Trileptal), lamotrigine (Lamitrin), pregabalin (Lyrica), and topiramate (Topamax) from Novartis Pharma, Germany, GlaxoSmithKline, Great Britain, Pfizer, Great Britain, and Janssen-Cilag, Belgium, respectively. All drugs were suspended in 1% solution of Tween 80 (Sigma, St. Louis, MO, USA), prepared freshly on each day of tests, and administered intraperitoneally in a volume of 10 ml/kg of body weight 30 (oxcarbazepine), 60 (sotalol and lamotrigine), 60 (topiramate), and 120 min (pregabalin) before the tests.

### Maximal electroshock seizure test in mice

The maximal electroshock (MES) test is a widely used preclinical model of tonic–clonic seizures [13]. Step-by-step procedures were described previously by Borowicz et al. [14]. The antielectroshock activity of antiepileptic drugs applied alone and in combinations with sotalol was determined as their ability to protect 50% of mice against tonic hindlimb extension induced by 25 mA electric current delivered by ear-clip electrodes. The dose–response curves were constructed based on the percentage of mice protected and the respective median effective doses (ED_50_ values in mg/kg) were evaluated [15].

### Chimney test

Possible motor coordination impairment induced by sotalol and its combinations with second-generation antiepileptic drugs was determined in the chimney test of Boissier et al. [16]. Methodology of this test was profoundly described in previous articles [14]. Antiepileptics were administered alone, at doses equal to their ED_50_ values, or in combinations with sotalol (100 mg/kg).

### Step-through passive-avoidance task

The step-through passive-avoidance test, referring to natural aversion of rodents to lighted places, was used as a measure of long-term memory. The task was previously outlined in detail by Borowicz et al. [14]. In the present study, however, a manual, but fully automated apparatus was used with specific hardware and software features [Multi Conditioning System (MCS), TSE Systems GmbH, Bad-Homburg, Germany]. The MCS software features are compliant with the Good Laboratory Practice. The apparatus allows entire isolation of animals from external stimuli that may interfere with their behavior. Therefore, the test results are more reliable when compared to the manual method. Thanks to a camera placed inside the chamber, MCS enables continuous observation of the animal's behavior on the monitor. A punishing electrical stimulus (0.3 mA for 2 s) was triggered in the dark compartment by rods in a greed floor.

Like in the chimney test, the antiepileptic drugs were applied alone at their ED_50_s or in combinations with sotalol (100 mg/kg). The results were presented as medians (with 25 and 75 percentiles) of time needed by animals to enter the dark box. Control animals (remembering an aversive electrical stimulus) did not enter the dark compartment within 180 s.

### Measurement of brain concentrations of antiepileptic drugs

Brain concentrations of four second-generation antiepileptic drugs (oxcarbazepine, lamotrigine, pregabalin, and topiramate) were determined by fluorescence polarization immunoassay. The pharmacological activity of oxcarbazepine is primarily exerted through the 10-monohydroxy metabolite (MHD). Since oxcarbazepine is rapidly reduced to MHD, the brain levels of this metabolite were actually measured.

The control groups were administered one of the antiepileptic drugs and saline. The examined groups received the combination of the respective antiepileptic drug and sotalol (100 mg/kg). Decapitation and brain removal took place at times scheduled for the MES test (at the peak of the antielectroshock action of antiepileptic drugs). Next, the brains were weighed and homogenized by Ultra Turax T8 homogenizer (IKA, Staufen, Germany) with Abbott buffer (2:1 vol/weight). Homogenates were centrifuged at 10,000 *g* for 15 min. Antiepileptic drug concentrations in supernatants (75 μl) were assessed by an Architect c4000 clinical chemistry analyzer (Abbott Laboratories Poland) and expressed as means ± SD of at least eight determinations in micrograms per milliliter.

### Measurement of brain concentrations of sotalol

Brain concentrations of sotalol were measured by liquid chromatography–mass spectrometry determination. The samples of brain homogenates were mixed with cold 1:1 v/v methanol:ethanol at 1:3 v/v ratio, vortexed, placed at − 20 °C for 15 min, vortexed again, and centrifuged at 4 °C for 5 min. Supernatants were subjected to liquid chromatography–mass spectrometry analysis (LC–MS). An Agilent Technologies liquid chromatograph 1290 Infinity series coupled to an Agilent Technologies quadrupole-time of flight mass spectrometer 6550 iFunnel LC/QTOF equipped with a Jet Stream Technology ion source was employed. Separations were carried out using a Zorbax Extend C18 RRHT 2.1 × 100 mm 1.8 μm column and water and acetonitrile, both with the addition of 0.1% v/v formic acid as mobile phases. The HRMS spectra were acquired in the positive polarity at the range of 100–1000 *m*/*z*. Internal mass calibration was enabled, and two reference ions of *m*/*z* 121.0509 and 922.0058 were used, to ensure mass measurement accuracy < 1 ppm. Agilent Technologies Mass Hunter software, B.10 for data acquisition and B.07 for processing, was utilized. The ions of m/z 273.1267 and 255.1162, attributed to [M + H]^+^ and [M-H2O + H]^+^, respectively, were extracted and merged to produce chromatograms for sotalol. EIC peaks were integrated and peak areas, which are proportional to analyte concentrations, were reported.

### Statistical analysis

The ED_50_ values with their respective 95% confidence limits were calculated using computer log-probit analysis according to Litchfield and Wilcoxon [15]. The standard errors of measurement (SEMs) of the mean values were assessed on the basis of confidence limits and the ED_50_ values were compared with one-way analysis of variance (ANOVA) followed by the post hoc Tukey test.

The Fisher’s exact probability test was used to analyze qualitative variables from the chimney test. The non-parametric Kruskal–Wallis test was applied for statistical assessment of the results obtained in the passive-avoidance task.

Brain concentrations of antiepileptic drugs and sotalol were evaluated using the unpaired Student’s *t* test. The significance level was set at *p* ≤ 0.05.

## Results

### Electroconvulsive threshold

Sotalol administered at the dose range of 60–100 mg/kg did not significantly influence the electroconvulsive threshold. The control value was 6.0 ± 0.52 mA [*F*(2.45) = 1.142; *p* = 0.3281; data not shown in tables].

### Maximal electroshock test

**Fig. 1:**
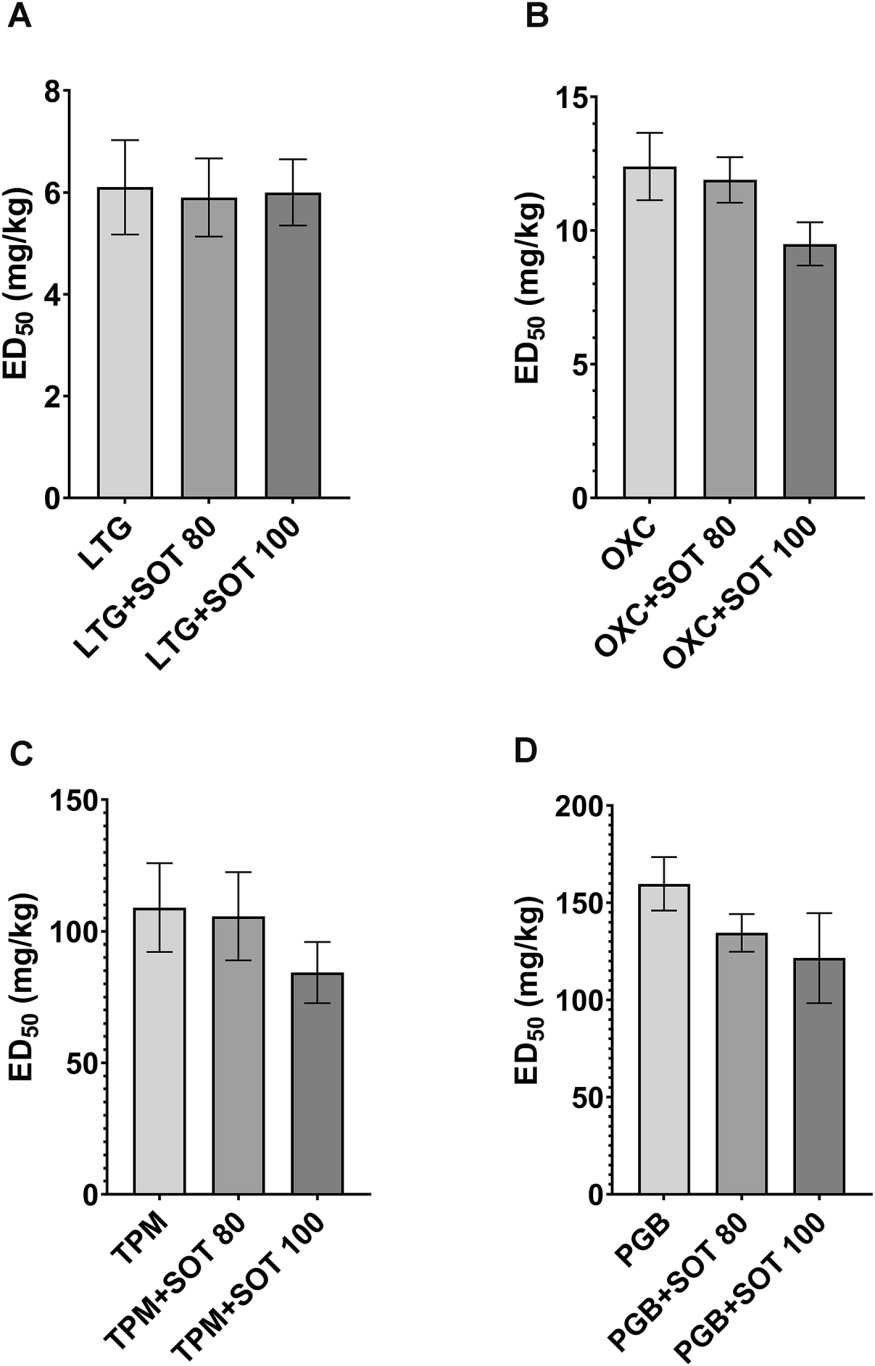
Effects of sotalol (SOT) on the anticonvulsant action of A. lamotrigine (LTG), B. oxcarbazepine (OXC), C. topiramate (TPM), and D. pregabalin (PGB) against maximal electroshock-induced seizures in mice. Data are presented as median effective doses (ED_50_ dose with SEM values) at which antiepileptic drugs alone and in combinations with SOT protected 50% of animals against seizures

In further experiments, sotalol applied at two doses (80 and 100 mg/kg), both ineffective in the electroconvulsive threshold test, was combined with antiepileptic drugs. Sotalol did not influence the antielectroshock action of lamotrigine [*F*(2.69) = 0.01494; p = 0.9852], oxcarbazepine [*F*(2.69) = 2.433; *p* = 0.0953], pregabalin [*F*(2.77) = 0.8784; *p* = 0.4196] or topiramate [*F*(2.83) = 0.3958; *p* = 0.6744]. The ED_50_ values for lamotrigine [6.1 (4.8–78) mg/kg], oxcarbazepine [12.4 (10.2–15.1) mg/kg], pregabalin [159.7 (138.0–184.8) mg/kg], and topiramate [109.00 (82.4–144.4) mg/kg] were not significantly changed by sotalol. Detailed data are listed in Fig. 1.

### Chimney test and passive-avoidance task

Sotalol and its combinations with antiepileptic drugs used in this study did not affect motor performance or long-term memory in mice, except for pregabalin, which applied alone (121.5 mg/kg) significantly impaired these parameters. In the case of combinations of sotalol with topiramate or pregabalin some tendency to compromise memory was observed. Therefore, the statistical parameters from Kruskal–Wallis test are presented as follows: 1. for sotalol + lamotrigine H = 8.211; N_1_ = 10, N_2_ = 10, N_3_ = 9, N_4_ = 10, *p* = 0.0418, 2. for sotalol + oxcarbazepine H = 8.414; N_1_ = 10, N_2_ = 10, N_3_ = 10, N_4_ = 10, *p* = 0.0382, 3. for sotalol + topiramate H = 9.247, N_1_ = 10, N_2_ = 10, N_3_ = 10, N_4_ = 8, *p* = 0.0262, and 4. for sotalol + pregabalin H = 11.52; N_1_ = 10, N_2_ = 10, N_3_ = 10, N_4_ = 9, *p* = 0.0092 (Table 1).

**Table 1:** Effects of acutely administered sotalol, antiepileptic drugs, and their combinations on motor performance and long-term memory in mice

Drugs and doses (mg/kg)	Animals impaired (%)	Retention time (s)
Vehicle	0	180 (176; 180)
SOT (100.0)	0	96.5 (16; 180)
LTG (6.0)	0	180 (180; 180)
LTG (6.0) + SOT (100.0)	0	180 (69; 180)
OXC (9.5)	0	180 (180; 180)
OXC (9.5) + SOT (100.0)	0	180 (162; 180)
TPM (84.3)	10	180 (180; 180)
TPM (84.3) + SOT (100.0)	0	36 (29.5; 148.5)
PGB (121.5)	33	37 (13; 123)**
PGB (121.5) + SOT (100.0)	10	67 (23; 87)

Data are expressed as percentage of animals that failed to perform the chimney test and as median retention time (with 25th and 75th percentiles) during which the animals avoided the dark compartment in the step-through passive-avoidance task. Statistical analysis of data obtained from the chimney test was calculated using the Fisher’s exact probability test, whereas the results from the step-through passive-avoidance task were analyzed using the non-parametric Kruskal–Wallis ANOVA test followed by the Dunn’s post hoc test. *LTG *lamotrigine, *OXC *oxcarbazepine, *TPM *topiramate, *PGB *pregabalin, *SOT *sotalol

**p < 0.01 vs vehicle

### Brain concentrations of antiepileptic drugs and sotalol

Sotalol (100 mg/kg) significantly decreased the brain concentration of lamotrigine, increased those of oxcarbazepine and topiramate, and did not affect the level of pregabalin (Table 2).

**Table 2:** Effects of acutely administered sotalol on the brain concentrations of antiepileptic drugs in mice

Treatment (mg/kg)	Brain concentration (µg/ml)	Statistics
LTG (6.0) + vehicle	0.21 ± 0.03	
LTG (6.0) + SOT (100.0)	0.16 ± 0.04**	*t*_18_ = 3.058, *p* = 0.0068
OXC (9.5) + vehicle	0.10 ± 0.02	
OXC (9.5) + SOT (100.0)	0.17 ± 0.04***	*t*_18_ = 4.856, *p* = 0.0001
TPM (84.3) + vehicle	10.59 ± 0.36	
TPM (84.3) + SOT (100.0)	11.57 ± 0.60***	*t*_18_ = 4.403, *p* = 0.0003
PGB (121.0) + vehicle	1.78 ± 0.31	
PGB (121.0) + SOT (100.0)	1.70 ± 0.22	*t*_18_ = 0.6784, *p* = 0.5061

Results are presented as the means ± SD of at least eight determinations. Statistical analysis of the brain concentrations was performed using the unpaired Student’s *t* test. *LTG *lamotrigine, *OXC *oxcarbazepine, *TPM *topiramate, *PGB *pregabalin, *SOT *sotalol

**p < 0.001, ***p < 0.001 vs control (the respective antiepileptic drug + vehicle)

On the other hand, no antiepileptic drug influenced the brain concentration of sotalol in mice (Table 3).

**Table 3:** Effects of acutely administered antiepileptic drugs on the brain concentrations of sotalol in mice

Treatment (mg/kg)	Brain concentration (peak area)	Statistics
SOT (100.0) + vehicle	3,569,521.4 ± 1,297,099.4	
SOT (100.0) + LTG (6.0)	3,347,690.9 ± 541,686.5	*t*_18_ = 0.499, *p* = 0.6238
SOT (100.0) + OXC (9.5)	3,582,523.5 ± 653,593.8	*t*_18_ = 1.311 *p* = 0.2064
SOT (100.0) + TPM (84.3)	3,031,708.1 ± 799,169.1	*t*_18_ = 1.116, *p* = 0.279
SOT (100.0) + PGB (121.0)	4,068,960.9 ± 817,354.1	*t*_18_ = 1.03, *p* = 0.3166

Results are presented as the means ± SD of at least eight determinations. Statistical analysis of the brain concentrations was performed using the unpaired Student’s *t* test. *LTG *lamotrigine, *OXC *oxcarbazepine, *TPM *topiramate, *PGB *pregabalin, *SOT *sotalol

## Discussion

The results presented herein showed that a single administration of sotalol did not influence the antielectroshock action of lamotrigine, oxcarbazepine, pregabalin, and topiramate. This finding seems advantageous from the preclinical point of view and supports the opinion that the second-generation antiseizure drugs rarely interact with other medications. Although the antiepileptics tested in this study did not change the brain concentrations of sotalol, this antiarrhythmic drug significantly decreased the brain level of lamotrigine (by 23.8%) and increased the levels of oxcarbazepine (by 70%) and topiramate (by 9.25%). In our previous study, sotalol did not influence the brain concentrations of some traditional antiepileptics, i.e., valproate, carbamazepine, phenytoin, and phenobarbital. Therefore, the observed enhancement of the antielectroshock properties of valproate and phenytoin was likely to be pharmacodynamic in nature [17]. The question than arises, why pharmacokinetic interactions revealed in the present study (particularly in the case of oxcarbazepine) did not translate into pharmacodynamic effects. The likely explanation is that pharmacokinetic events masked real synergism between sotalol and lamotrigine and antagonism between sotalol and oxcarbazepine or topiramate.

Sotalol is primarily a β-blocker exhibiting class III antiarrhythmic properties. A medication with a dual mechanism of action may offer some advantages as a kind of one-pill two-drug therapy, especially that β-blockers are recommended to be co-applied with antiarrhythmic drugs [18]. Moreover, blockade of β-adrenoceptors may facilitate the antiseizure activity. In contrast, blocking of KCNH2 potassium channels can lead to opposite effects, which is supported by the finding that mutations of KCNH2 channels are a potential common background of epilepsy and arrhythmias related to long QT-2 syndrome [19]. Considering these data, it seems interesting whether sotalol may be really a better choice than traditional β-blockers in patients with epilepsy.

Sotalol is characterized by low brain penetration. Nevertheless, it crosses the blood–brain barrier better than atenolol and achieves the brain concentration of approximately 0.65 μg/ml when administered into the vertebral vein at the dose of 1 mg/kg. Moreover, the metabolites of sotalol reach the level of about 1.65 μg/ml. The same antiarrhythmic drug given to the femoral vein exhibited much lower brain/plasma uptake. The respective concentrations did not exceed 0.1 μg/ml [20]. Nevertheless, we assumed that sotalol, applied at higher doses and reaching higher brain concentrations, may be sufficient to influence the seizure processes.

According to the data obtained in the present study, sotalol per se did not affect the electroconvulsive threshold, which fits well its dual mechanism of action. Amiodarone, another III class antiarrhythmic drug, was also found inactive [21]; however, its anticonvulsant properties were observed against pentetrazole- and caffeine-induced convulsions in mice [11]. On the other hand, numerous β-blockers penetrating the blood–brain barrier exhibited anticonvulsant action in various experimental seizure models. Taking into consideration the electrically evoked seizures alone, propranolol, metoprolol, acebutolol, but not timolol, showed protective effects in the electroconvulsive threshold test [22, 23, 25].

Moreover, sotalol did not affect the antielectroshock action of lamotrigine, oxcarbazepine, pregabalin, and topiramate. Regarding classical antiepileptics, sotalol enhanced the action of valproate and phenytoin, but not that of carbamazepine and phenobarbital. Amiodarone, a multi-blocker of potassium, sodium, and calcium channels, enhanced the antielectroshock action of carbamazepine, but remained without effect on the action of valproate, phenytoin, and phenobarbital. According to our unpublished data, amiodarone also enhanced the action of pregabalin and oxcarbazepine, but not that of lamotrigine or topiramate against the maximal electroshock seizures in mice. Moreover, some classical β-blockers potentiated the antielectroshock action of antiepileptic drugs and other anticonvulsant substances. For instance, propranolol and metoprolol enhanced the action of valproate, diazepam, dizocilpine (MK-801), an NMDA receptor antagonist, and 1-(4-aminophenyl)-4-methyl-7,8-methylenedioxy-5H-2,3-benzodiazepine (GYKI 52466), a non-NMDA (AMPA/kainate) receptor antagonist. Acebutolol enhanced the effect of valproate and GYKI 52466, whereas atenolol did not affect the action of either antiepileptic drugs or glutamate receptor antagonists [23, 24]. Furthermore, in the methodologically similar increasing-current electroshock seizure test in mice yet not in the maximal electroshock test, carvedilol (a nonselective alpha and β-blocker) potentiated the effect of gabapentin [25].

Since sotalol is characterized by low lipophilicity and blood–brain permeability, the question arises, whether effects of this antiarrhythmic drug on seizure phenomena could be an indirect consequence of its hypotensive action. Our results and the results of Luchowska et al. [23, 24] do not seem to confirm this hypothesis. The most lipophilic β-blockers, reaching the highest brain concentrations (propranolol and metoprolol), were found to have the greatest effect of the anticonvulsant action of other drugs. Acebutolol exhibited a moderate effect, while the most hydrophilic atenolol did not affect the action of any tested medication. In terms of lipophilicity (and brain concentrations), sotalol is usually placed between acebutolol and atenolol. Moreover, cardiovascular efficacy of metoprolol and its influence on blood pressure are comparable to those of atenolol and sotalol [20, 26]. Therefore, it seems that the influence of β-blockers on antiepileptic drugs is due to the central rather than hypotensive action. It is worth adding that the hypotensive effects of β-receptor antagonists are due to peripheral and not central mechanisms [20].

Unfortunately, the clinical data regarding the action of sotalol or amiodarone in patients with epilepsy are not available. On the other hand, the clinical data on the influence of β-blockers are very scarce. The only two reports about the anticonvulsant action of propranolol in patients with drug-resistant chronically unstable generalized epilepsy and startle induced epileptic seizures were published 25 years ago [27, 28]. In our opinion, based on the pharmacodynamic results obtained in the present study, sotalol can be used on par with β-blockers as an arrhythmic drug in patients with epilepsy. Like in the case of other antiarrhythmics, interactions with antiepileptic drugs, particularly these traditional, can be expected. However, their type cannot be predicted based on theoretical premises.

In the present study, we observed some pharmacokinetic drug interactions. Sotalol decreased the brain level of lamotrigine and increased the levels of oxcarbazepine and topiramate, which is quite surprising since sotalol did not affect the brain levels of valproate, carbamazepine, phenytoin, or phenobarbital measured in the previous study [17]. Interestingly, sotalol does not bind to plasma proteins and is not metabolized, which markedly reduces possibility of pharmacokinetic interactions [29]. Furthermore, lamotrigine and oxcarbazepine (MHD) are not highly bound to plasma proteins (in about 55 and 40%, respectively), while pregabalin does not bind to plasma proteins at all. Therefore, clinically significant interactions with other drugs through competition for protein binding sites are unlikely. Topiramate and pregabalin are not extensively metabolized and are eliminated primarily by renal excretion in unchanged forms. The effects of lamotrigine on hepatic oxidase isozymes have not been systematically evaluated. Oxcarbazepine was the only drug reported to inhibit CYP2C19 and induce CYP3A4/5 with potential impact on plasma concentrations of other drugs [1, 30–32]. The above considerations do not authorize, however, the pharmacokinetic interactions revealed in the present study. Nevertheless, it should be remembered that the pharmacokinetic data presented concern the human metabolism. The reliable animal parameters, specifically in mice, are not available.

As regards neurotoxic undesired effects, sotalol and its combinations with antiseizure drugs used in this study did not significantly impair motor performance or long-term memory in mice, which is similar to previous findings. Although, a clear tendency toward memory impairment can be seen at the first sight in the case of treatment with sotalol alone and its combinations with pregabalin and topiramate, it did not reach the level of significance. Pregabalin applied alone was the only one to markedly compromise memory in mice. However, such an effect can be to some extend due to the analgesic properties of pregabalin, thanks to which animals felt less the aversive stimulus. Similar results in terms of undesired effects were observed in the previous study [33]. Sotalol, first-generation antiepileptic drugs, and combinations of sotalol with antiepileptics did not cause any motor or long-term memory deficits. However, any direct comparison between the two studies with sotalol cannot be translated into previous findings because of methodological differences in the step-through passive-avoidance test. It is worth mentioning that in the older manual method, none of traditional antiepileptic drugs (valproate, carbamazepine, phenytoin or phenobarbital) affected the aversive behavior in mice. In MCS, valproate and carbamazepine significantly decreased the mean time spent in the illuminated box [34]. It should be assumed that the MCS results are more reliable than those acquired from the manually performed task.

Finally, it is worth underlining that the recommended starting dose of sotalol in clinical practice is 240 or 320 mg/day and the maximal dose is 640 mg/day [35]. In our study, we did not measure the plasma concentrations of sotalol; therefore, a comparison with respective therapeutic concentrations in human plasma was not possible. Nevertheless, there are equations for conversing mouse doses into human doses. In our study, sotalol was applied at two doses of 80 and 100 mg/kg, which translated to the human body (70 kg) give approximately 6.4–8.1 mg/kg or 454–567 mg per dose [36]. Noteworthy, despite relatively high doses of the antiarrhythmic agent used in animals, no significant neurotoxic effects were observed. However, further analysis of electrophysiological and inotropic implications of the combined treatment with sotalol and antiepileptic drugs is strongly advised.

Summing up, our findings revealed that sotalol, although blocking the potassium channels and potential proconvulsant effects, did not influence the electroconvulsive threshold and the antielectroshock action of the antiseizure drugs used in the present study. Additionally, no significant neurotoxic adverse effects were observed. In this sense, sotalol can be considered a relatively safe antiarrhythmic drug in patients with epilepsy treated with lamotrigine, oxcarbazepine, pregabalin, or topiramate However, our preclinical data, including the pharmacokinetic interactions disclosed, should be confirmed in other seizure models and in clinical conditions.
